# Resuscitation from Chloroform Syncope

**Published:** 1879-07

**Authors:** E. F. Starr

**Affiliations:** Nacoochee, Ga.


### RESUSCITATION FROM CHLOROFORM SYNCOPE.

By E. F. STARR, M.D., Nacoochee, Ga.

The credit of having discovered and first put into practice the best method of resuscitation from the alarming effects of chloroform, ether, etc.—that is, the threatened or actual state of syncope which sometimes attends the use of these articles—is due, I believe, to Nelaton, the great French surgeon. Fortunately, this evil effect does not often result from the legitimate use of these things; yet, as long as it is liable to occur at all, the most efficient way of counteracting it, or of obtaining recovery from it, should be familiar to every one who ventures to administer these renowned anæsthetics. Probably I could not make this fact more impressive or apparent, nor bring better to view the importance of a knowledge of, and familiarity with, this method of proceeding, than by relating an illustrative case.

I had occasion, two years ago, to do a little surgery on the foot of a youth seventeen years of age, to remedy a deformity caused by a burn while he was a child. The operation was to be a little severe, and chloroform was desired. He was laid upon a table in an open piazza, and the chloroform carefully administered. From some cause (I believe his mother told me she had known him to have fainty spells,) I was a little apprehensive in regard to the effects the chloroform might have upon him, and soon found he did not get along well under it. The inhalation was suspended for a little time, and the pillow taken from under his head. This gave some relief, and the inhalation was resumed; but soon again the symptoms were not good. The breathing was imperfect, the pulse flagging, etc., and the operation had to be suspended, to pay attention to the induced condition of the patient. Soon he vomited, and the quantity ejected showed that his stomach was fuller at the commencement of the proceedings than it shoud have been, but, contrary to my expectations, the emptying of the stomach, and the exertion necessary to do it, did not improve his condition much. The operation was completed while a close watch was kept upon the patient.

The chloroform was discontinued as soon as practicable; consciousness soon returned, but he did not at the same time recover from the depressing effect of the anæsthetic, notwithstanding the administration of stimulants, cold sprinkling, fanning, etc. He was carried into the house and laid upon a convenient little bunk, which his father had prepared for him, and stimulants and the various ordinary means continued. His pulse was slow—very slow, and his breathing difficult and unsatisfactory, and he would exclaim occasionally, "Oh! I am so tired." At last, finding that the usual means were not sufficient to restore him, and having Nelaton's method in mind, I had the foot of his bed raised, but not enough; it did not answer. I then ordered it elevated and fixed on the back of a chair at an angle of thirty-five or forty degrees, with no pillow under his head, when almost immediately all the alarming symptoms disappeared; the breathing became easy, the pulse reacted, the countenance resumed its natural expression—in short, the patient in a few minutes was restored to a normal condition.

In this case there was no actual suspension of animation, of pulse, nor breathing, but an alarming approach to these; and if the inhalation had been persisted in, without intermission and careful attention to the patient's condition, it is more than probable there would have been complete syncope. As it was, he was evidently restored by the pressure of blood upon the brain and upper cord, induced by the reversed position—the force of gravitation supplied the brain and medulla with blood enough to stimulate them to action, or to arouse their driving power. A slight reversion will not do—the heels must be far above the head—it must be thorough and persistent.

Dr. J. Marion Sims has related that "Ten years ago, there was a story prevalent in Paris that M. Nelaton had derived the hint of reversing the body in chloroform poisoning from a discovery accidentally made by his little son—that the little boy had killed some mice with chloroform; that without thought or reason he had taken up a dead mouse by the tail and was twirling it around, when to his surprise it began to manifest signs of life, and recovered entirely, while the mice left lying were dead, and that the great surgeon was thus taught a great lesson. This is a very pretty story as it is, and it seems a pity to spoil it. When in Paris I called to see young Nelaton, (now a student of medicine,) and I asked him for the facts of the mouse story. He said that when they lived on the Quai Voltaire the house was infested with mice; that great numbers were caught in traps almost daily; that he was in the habit of killing them with chloroform by covering the trap with a napkin and pouring the chloroform on it, and that his only idea was an easy death for the mice. One day his father accidentally came into the room, and seeing the dead mice, he told his son if he would take one by the tail and hold it with the head downwards it would revive, while the others would not. He did this and found it was true."

For a graphic and detailed narrative of an interesting and extreme case of resuscitation from chloroform syncope, see an extract in the *American Journal of Medical Sciences* for October, 1874, from a paper read by Dr. Sims before the British Medical Association.

Usually, the mode of action of chloroform and ether seems to be that of suspending the functions of the sensory and motor system of nerves—at least the voluntary motors—and leaving the sympathetic or that of animal life or involuntary, still active; but when these latter are overcome, as they are sometimes liable to be, there is suspension of all the functions—of the action of the heart and respiratory muscles, of the circulation of the blood, and consequently of all animation, just as there is in syncope; but now, if, while the blood is warm and fresh, it is made, by the force of gravitation, to rush upon the brain and upper cord, these will be so stimulated as to recover their driving power and produce resuscitation.

The effects of chloroform are often alluded to as poison in the blood. I do not regard it as such, pure and simple, but rather as simply a suspender of the nervous influence; for as long as as the heart beats on and the breathing continues, the blood is not poisoned, and there is no harm done.

As to the comparative safety of chloroform and ether, I am of the opinion there is not so much difference as some believe; that ether is not absolutely safe, and furthermore, that it is scarcely probable any efficient anæsthetic agent will ever prove to be entirely free from accidents under extensive application.

For chloroform I can say, that of the many thousands of administrations of this substance, whether prudently or carelessly, whether cautiously or recklessly, of which I was cognizant during the late war, I did not know of a single fatality on account of it; nor have I ever before or since witnessed a fatal case. In view of this question, some one has well remarked that who ever desires the benefits of anæsthesia, must assume the little risk that may pertain to whatever agent that may be employed.

